# Advancement through epidermis using tape stripping technique and Reflectance Confocal Microscopy

**DOI:** 10.1038/s41598-019-48698-w

**Published:** 2019-08-21

**Authors:** Caroline Meyer Olesen, Christine Sofie Krohn Fuchs, Peter Alshede Philipsen, Merete Hædersdal, Tove Agner, Maja-Lisa Clausen

**Affiliations:** Department of Dermatology, Bispebjerg Hospital, University of Copenhagen, Bispebjerg Bakke 23, 2400 Copenhagen, NV Denmark

**Keywords:** Confocal microscopy, Confocal microscopy, Skin diseases, Skin diseases

## Abstract

The tape stripping technique is increasingly used in research regarding skin barrier function. However, number of tape strips varies between studies, and literature considering advancement into stratum corneum/epidermis in relation to number of tape strips is scarce. The aim of this pilot study was to assess the advancement through epidermis using tape stripping technique in healthy volunteers. A total of ten healthy volunteers were included. From all volunteers 0, 5, 15 and 35 consecutive tape strips (D-squame) were taken from four adjacent skin areas on the middle volar forearm, followed by Reflectance Confocal Microscopy (RCM) of the four areas to assess epidermal thickness. Squame Scan was used to determine amount of protein removed. Stratum corneum was completely removed in all volunteers after 35 tape strips. Advancement into epidermis was predominantly achieved by the first 15 tape strips, removing 25% of the total epidermis, whereas 35 tape strips removed 33% of epidermis. Protein removal per tape decreased with increasing depth. Information on advancement into the epidermis according to number of tape strips taken, is a significant step forward. The possibility to obtain samples from different layers of epidermis may lead to an improved understanding of skin barrier properties.

## Introduction

During the last decade the tape stripping technique for removal of stratum corneum has been increasingly used in research regarding skin barrier function. The method has been applied for experimentally induced impairment of barrier function^1^, for penetration studies^2,3^ and more recently also for assessment of epidermal biomarkers in inflammatory skin diseases^4,5^. With respect to the latter, the advantages, as compared to skin biopsies, are many, since analysis of biopsies may be mixing of epidermal and dermal structures, and even include subcutaneous tissue. Furthermore, when using skin biopsies, there is a lack of possibility for follow-up within the same skin area, and last but not least the problem with discomfort and scarring that biopsies may cause to patients and volunteers.

With respect to tape stripping, a consistent method has been developed, using a specialized tape (D-squame tape), and standardized pressure^4,6–10^. In spite of this effort to standardize the method, number of strips taken varies between studies. After removal of 30–40 strips the skin is described as shinny and red, and it is generally agreed that the stratum corneum has been removed^9,11–13^. Literature on this aspect is, however, scarce, and biopsies taken after removal of strips do not display the thickness of epidermis clearly. This uncertainty of how deep into epidermis the method advances has posed a bias when using tape stripping for investigation of skin immunology and epidermal biomarkers, as information on the exact epidermal location of collected cells and substances is essential for the interpretation of results. Recently, Reflectance Confocal Microscopy (RCM) was shown to be a suitable method for assessment of the epidermal thickness, and to correlate with histological findings^13^. The aim of this study was to assess the advancement through epidermis in healthy volunteers by use of D-squame tape stripping technique with standardized pressure monitored by RCM.

## Results

A total of ten healthy volunteers, seven women and three men, mean age 31 (range 26–46), participated in the study. At baseline mean values of transepidermal water loss (TEWL) was 8.5 g/m^2^/h (interquartile range (IQR): 6.8–12.9) and mean pH was 5.91 (IQR 5.60–6.58).

### Epidermal thickness

The thickness of epidermis as measured by RCM without tape stripping demonstrated very homogenous data, with little inter-individual differences (Table 2). Median epidermal thickness before tape stripping (T0) was 56.9 µm (Table 1).

**Table 1:** Epidermal thickness and protein removal.

*Healthy volunteers*	Epidermal thickness (µm) median (IQR) *(CV%)*	Percent removed of total epidermis	Protein removed (µg/cm^2^) median (IQR) *(CV%)*	Number of participants with stratum corneum removed
T0	56.9	0	0	0/10
	*(54.1–61.5)*			
	*−9%*			
T5	50.5	11.30%	85.6	0/10
	*(47.4–52.2)*		*(49.9–110.3)*	
	*−7%*		*−50%*	
T15	42.9	25.00%	220.6	01-Oct
	*(42.0–44.8)*		*(70.3–261.7)*	
	*−6%*		*−49%*	
T35	37.8	32.70%	305.2	10-Oct
	*(35.6–40.6)*		*(99.8–392.6)*	
	*−9%*		*−49%*	

Epidermal thickness (µm), percent removal of epidermis (%), and protein removed (µg/cm^2^) before tape stripping (T0), after 5 tape strips (T5), after 15 tape strips (T15) and after 35 tape strips (T35). Results are shown as median (interquartile range (IQR)) as well as coefficient of variance (CV%).

**Table 2:** Epidermal thickness.

Healthy Volunteers	Epidermal thickness (µm) T0	Epidermal thickness (µm) T5	Epidermal thickness (µm) T15	Epidermal thickness (µm) T35
HV1	61,72	56,44	45,21	42,17
HV2	56,89	49,79	42,67	39,13
HV3	54,35	50,80	44,70	43,18
HV4	53,34	47,76	43,18	36,07
HV5	64,00	54,86	49,79	36,58
HV6	61,47	51,31	42,67	37,55
HV7	56,39	50,78	39,12	31,50
HV8	46,74	46,24	41,67	34,04
HV9	56,90	45,21	44,16	38,09
HV10	58,41	50,29	42,16	40,13

Epidermal thickness (µm) for each healthy volunteer (HV), assessed before tape stripping (T0), after 5 tape strips (T5), after 15 tape strips (T15) and after 35 tape strips (T35).

**Figure 1:**
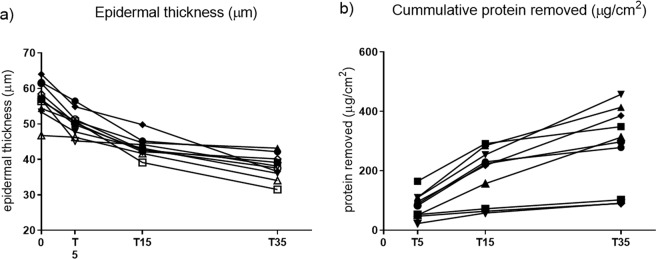
Epidermal thickness and cumulative protein removal. (**a**) Epidermal thickness (µm) assessed before tape stripping (T0), after 5 tape strips (T5), after 15 tape strips (T15) and after 35 tape strips (T35). A significant reduction in epidermal thickness was found from T0 to T35. (**b**) Cumulative protein removed (µg/cm^2^) assessed after 5 tape strips (T5), after 15 tape strips (T15) and after 35 tape strips (T35). Assessment was performed on non-lesional skin of healthy volunteers on the volar side of the forearm.

**Figure 2:**
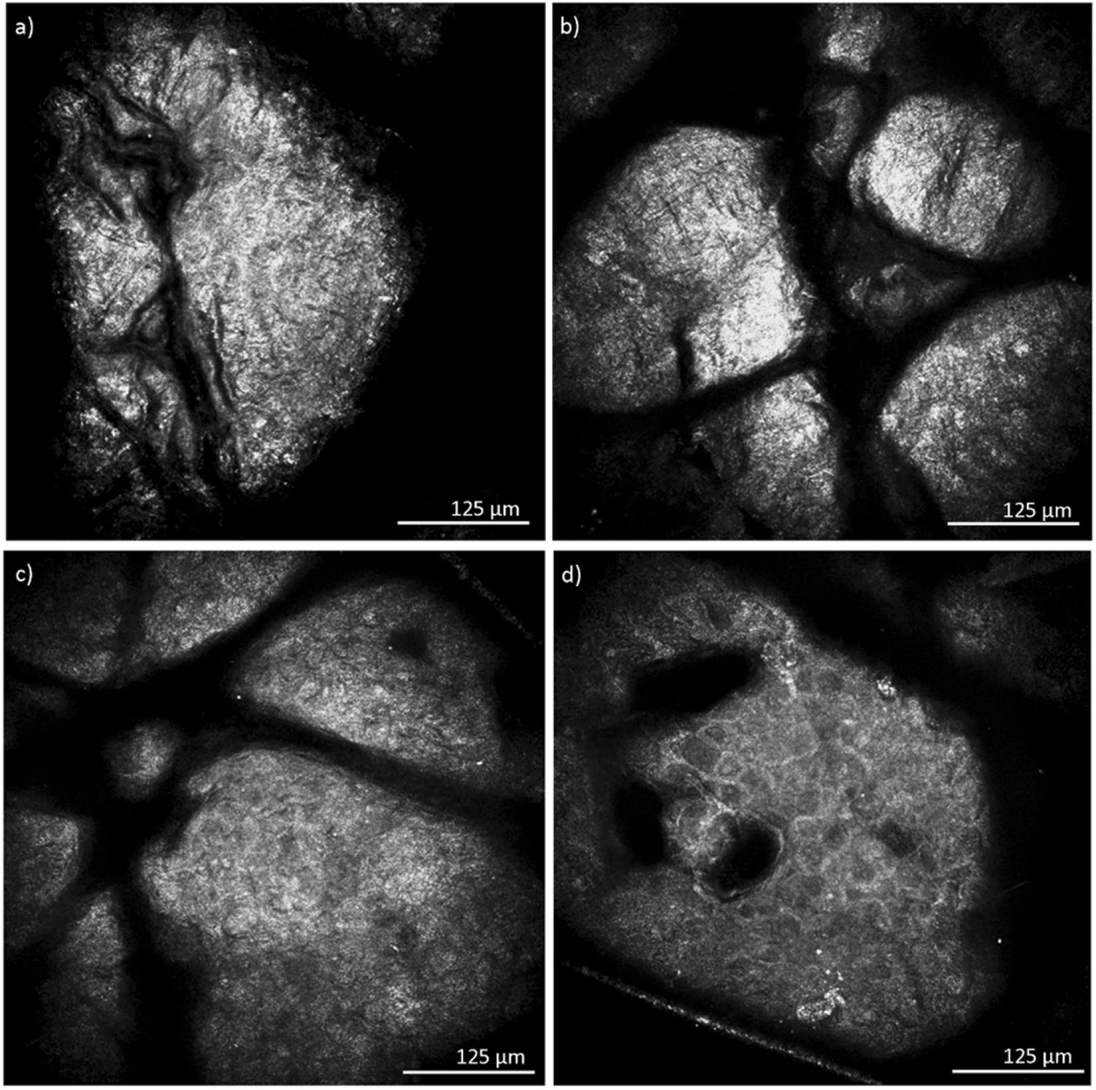
RCM images at 6 µm depths after 0, 5, 15 and 35 tape strips. **(a**–**d**) Horizontal RCM images at 6 µm from skin surface. (**a**,**b**) After 0 and 5 tape strips. Stratum corneum appears as a white, hyper reflective layer. (**c)** Parts of stratum corneum are still visible in the image, some areas present with a honeycomb pattern, corresponding to stratum granulosum. (**d)** After 35 tape strips, stratum corneum is completely removed, leaving only stratum granulosum visible.

There was a significant decrease in epidermal thickness from assessment at baseline to assessment after 35 tape strips (p = 0.001) (Fig. 1). Advancement towards the basement membrane was predominantly achieved by the first 0–15 tape strips, as tape 0–5 removed 1.3 µm/tape (1.0–1.9) (median (interquartile range (IQR)), whereas tape 6–15 removed 0.7 µm/tape (0.5–0.9) and the last twenty tapes from tape 16–35 removed 0.3 µm/tape (0.1–0.4). After five tape strips 11.3% of epidermis was removed, after 15 tape strips 25.0% of epidermis was removed and after 35 tape strips 32.7% of epidermis was removed (Table 1, Fig. 2).

Stratum corneum was completely removed after 35 tape strips in all volunteers as evaluated visually and by RCM. After five tape strips stratum corneum was removed in none of the volunteers, after 15 tape strips stratum corneum was removed in only one volunteer.

### Protein removal

**Figure 3:**
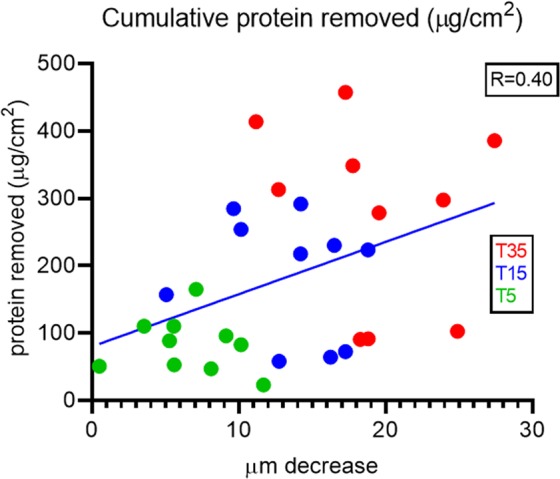
Correlation between protein removal and decrease in epidermal thickness. Correlation between protein removal (µg/cm^2^) and decrease in epidermal thickness (µm). Three samples from each participant are included: T5 = after 5 tape strips, T15 = after 15 tape strips and T35 = after 35 tape strips. Correlation (R = 0.40) was statistically significant (p = 0.038).

Protein removal per tape decreased with advancement through epidermis, with 17.1 µg protein/tape (10.0–22.1) (median (IQR)) on tape 1–5, 12.4 µg protein/tape (3.1–14.2) on tape 6–15 and 3.3 µg protein/tape (1.6–8.0) on tape 16–35, respectively, and showed great inter-individual variation (coefficient of variation (CV%) = 48–50). A correlation between protein removed and decrease in epidermal thickness was found (R = 0.40, p = 0.038) when analyzing all samples (Fig. 3).

### Transepidermal water loss

TEWL increased significantly after tape stripping (p = 0.002), with delta value from T0 – T35 being 31.8 g/m^2^/h (9.5–83.0) (median (IQR)).

## Discussion

The tape stripping technique has recently attracted much attention, since the method is a minimal invasive technique useful for sampling of epidermis and has a significant advantage to skin biopsies with respect to discomfort for patients and volunteers. Although increasingly used, the exact advancement into epidermis following tape stripping has remained unknown. In the present study this was explored by RCM, which clearly showed that the stratum corneum had been completely removed after 35 strips in all volunteers, with a removal of one third of the total epidermis. While 11% of the epidermis was removed after the first five tape strips, and 25% was removed after 15 tape strips, the additional 20 tapes did not advance to the same degree into the epidermis. Increasing cohesion between cells when advancing into the epidermis is likely to explain this as the first five tape strips caused a decrease in the epidermal thickness by 6.4 µm, whereas the last 20 tape strips decreased the epidermal thickness only by 4.4 µm. Increased cohesion in the deeper layers of epidermis has been reported in previous studies^14,15^. Although gender ratio in this study was skewed with 70% women and 30% men, it is not expected to influence the results, as previous studies have shown that gender does not influence tape stripping with respect to protein data^16^.

A correlation was found between protein removal and decrease in epidermal thickness. Interestingly, it seems as there is a larger variation in protein removal in the deeper layers (T15 and T35) compared to the upper layers (T5) (Fig. 3). This likely reflects individual differences in strength and structure of the epidermis, as some participants seem to have a skin that very easily strips off when visually evaluating the tapes, as compared to others. In addition, the Squame Scan only scans the middle area of the tape corresponding to 50% of the total tape, and accordingly, uneven attachment of the skin to the tape, might cause a poor correlation between protein determination and decrease in epidermal thickness.

Until now an important bias related to tape stripping has been the uncertainty of how deep into the epidermis the method reaches, and methods like visual assessment, measurement of TEWL and protein content have not proved sufficient for solving this problem. Previously the appearance of red glistening skin and/or significant increase in TEWL have been interpreted as the removal of stratum corneum. Using the RCM to follow the gradual tape stripping of stratum corneum, we here present new data illustrating step by step how far down into the epidermis the method reaches. These data are new and necessary basic data for future use of tape stripping in the investigation of the skin barrier.

An important limitation to the tape stripping method is that skin furrows may complicate an exact differentiation of different layers in the epidermis^17^. Furthermore, the method is aimed predominantly at the skin barrier (stratum corneum), as reaching deeper levels might be challenging.

## Conclusion

Tape stripping is useful for skin barrier research and has the advantage over skin biopsies that it allows for collection of stratum corneum without scars and discomfort for the participant/patient. Results from the present study give a clear indication of advancement into epidermis according to number of tape strips, and open for the possibility to evaluate differences in skin barrier properties in different parts of the epidermis.

## Methods

### Participants

Healthy volunteers were recruited from the Department of Dermatology, Bispebjerg Hospital, Copenhagen. Topical application of emollients or other topical treatments was not allowed on the day of skin sampling.

### Tape stripping

Each participant had 0, 5, 15 and 35 consecutive tape strips (D-squame 3.8 cm^2^) (CuDerm) taken from four adjacent skin areas on the middle volar forearm (non-lesional skin). Each tape was pressed against the skin for 10 sec with standardized pressure (225 gr./cm^2^), using the D-squame pressurizer (CuDerm), before stripped from the skin. Localization for tape stripping was randomized from a planned system to four adjacent skin areas 4, 8, 12, 16 cm from the wrist, respectively.

### Reflectance confocal microscopy (RCM)

A commercially available reflectance confocal microscope (Vivascope Multilaser 1500®, Caliber ID Inc., Rochester, NY, USA) was applied in 658 nm mode. The RCM provides real-time horizontal images of the skin to a depth of 200–250 µm, which includes the entire epidermis and upper papillary dermis. In each test area, i.e. 0, 5, 15 and 35 tape strips respectively, 3 individual z-stacks stacks consisting of horizontal 500 × 500 µm optical sections were acquired at every 1.5 µm from skin surface to a depth of 82 µm, resulting in 53 images per z-stack.

RCM z-stacks were used to identify stratum corneum, stratum granulosum, stratum spinosum and the basement membrane; the latter with visible papillae in it. To assess epidermal thickness after 0, 5, 15, and 35 tape strips, the depth from the surface of the skin to the first visible papillae in the basement membrane was assessed in RCM images. To take variation of the papillae length into consideration, we decided that three papillae should be visible in the z-stack before measuring the depth. Measurements were performed in all three z-stacks from each test area and mean depth from skin surface to basement membrane represented epidermal thickness.

### Skin barrier function

Skin barrier function was assessed by measurements of TEWL, at the middle volar forearm before tape stripping and after 35 tape strips (DermaLab, Cortex Technology, Denmark). Participants adjusted to room temperature before TEWL measurements. Skin pH was measured at the middle volar forearm using a pH-meter (DermaLab, Cortex Technology, Denmark). All measurements were performed in triplicates, and mean values calculated.

### Protein content

To assess the amount of protein removed from the skin, each tape was scanned using Squame Scan 850 according to protocol^18^. In brief, each tape is scanned by the instrument, and optical absorption at 850 nm is measured. The protein content on each tape is then calculated from the optical density (OD) value using the standard formula: OD = 0.623x + 2.703^8,19^.

### Statistics

Wilcoxon matched-pairs signed rank test was used to test differences in epidermal thickness after 0, 5, 15 and 35 tape strips. Spearman r correlation was used to analyze correlation between decrease in epidermal thickness and protein removal. Statistical analyses were carried out using Prism 6 Graph Pad.

### Ethical considerations

This study was approved by the local ethics committee (De Videnskabsetiske Komiteer – The Capital Region of Denmark, project number H-1-2014-039) and the Danish Data Protection Agency (project number 01767 BBH-2012-019). All methods were carried out in accordance with relevant guidelines and regulations. All participants received oral and written information, and informed consent was obtained.

## Data Availability

The datasets generated during and/or analyzed during the current study are available from the corresponding author on request.

## References

[1] Jungersted JM, Hogh JK, Hellgren LI, Jemec GB, Agner T (2010). Skin barrier response to occlusion of healthy and irritated skin: differences in trans-epidermal water loss, erythema and stratum corneum lipids. Contact Dermatitis.

[2] Jacobi U, Meykadeh N, Sterry W, Lademann J (2003). Effect of the vehicle on the amount of stratum corneum removed by tape stripping. J Dtsch Dermatol Ges.

[3] Jakasa I, Verberk MM, Esposito M, Bos JD, Kezic S (2007). Altered penetration of polyethylene glycols into uninvolved skin of atopic dermatitis patients. J. Invest. Dermatol..

[4] Koppes SA (2016). Stratum Corneum Tape Stripping: Monitoring of Inflammatory Mediators in Atopic Dermatitis Patients Using Topical Therapy. Int. Arch. Allergy Immunol..

[5] Clausen ML, Slotved HC, Krogfelt KA, Agner T (2018). Measurements of AMPs in stratum corneum of atopic dermatitis and healthy skin-tape stripping technique. Sci. Rep..

[6] Breternitz M, Flach M, Prassler J, Elsner P, Fluhr JW (2007). Acute barrier disruption by adhesive tapes is influenced by pressure, time and anatomical location: integrity and cohesion assessed by sequential tape stripping. A randomized, controlled study. Br. J. Dermatol..

[7] Bashir SJ, Chew AL, Anigbogu A, Dreher F, Maibach HI (2001). Physical and physiological effects of stratum corneum tape stripping. Skin Res. Technol..

[8] Clausen ML, Slotved HC, Krogfelt KA, Agner T (2016). Tape Stripping Technique for Stratum Corneum Protein. Analysis. Sci. Rep..

[9] de Jongh CM, Verberk MM, Spiekstra SW, Gibbs S, Kezic S (2007). Cytokines at different stratum corneum levels in normal and sodium lauryl sulphate-irritated skin. Skin Res. Technol..

[10] Two AM (2016). The Cutaneous Microbiome and Aspects of Skin Antimicrobial Defense System Resist Acute Treatment with Topical Skin Cleansers. J. Invest. Dermatol..

[11] Kalia YN (2000). Normalization of stratum corneum barrier function and transepidermal water loss *in vivo*. Pharm. Res..

[12] Kropp PJ (1957). Examination of the epidermis by the strip method. IV. The melanocytes during a period of forced regeneration. J. Invest. Dermatol..

[13] Peppelman M, van den Eijnde WA, Jaspers EJ, Gerritsen MJ, van Erp PE (2015). Combining tape stripping and non-invasive reflectance confocal microscopy: an *in vivo* model to study skin damage. Skin Res. Technol..

[14] Loffler H, Dreher F, Maibach HI (2004). Stratum corneum adhesive tape stripping: influence of anatomical site, application pressure, duration and removal. Br. J. Dermatol..

[15] Chapman SJ, Walsh A, Jackson SM, Friedmann PS (1991). Lipids, proteins and corneocyte adhesion. Arch. Dermatol. Res..

[16] Jacobi U, Gautier J, Sterry W, Lademann J (2005). Gender-related differences in the physiology of the stratum corneum. Dermatology.

[17] van der Molen RG (1997). Tape stripping of human stratum corneum yields cell layers that originate from various depths because of furrows in the skin. Arch. Dermatol. Res..

[18] https://heilandelectronic.de/files/documents/SQM_Uman2_3.pdf.

[19] Voegeli R, Heiland J, Doppler S, Rawlings AV, Schreier T (2007). Efficient and simple quantification of stratum corneum proteins on tape strippings by infrared densitometry. Skin Res. Technol..

